# Supplementary material for: Multiplexed drug testing of tumor slices using a microfluidic platform
Source: NPJ Precis Oncol. 2020 May 19;4:12. doi: 10.1038/s41698-020-0117-y (PMC7237421; doi:10.1038/s41698-020-0117-y)
Supplement: Supplementary file 2 — Supplementary Materials [file 41698_2020_117_MOESM2_ESM.docx]

Long Title

**Multiplexed drug testing of tumor slices using a microfluidic platform**

Short Title

Microfluidic drug delivery to tumor slice cultures

**Authors**

L. F. Horowitz^1#,2,3^, A.D. Rodriguez^3^, Z. Dereli-Korkut^5^, R. Lin^3^, K. Castro^3^, A.M. Mikheev^2,5#^, R.J. Monnat Jr.^1,3,4^, A. Folch*†^3^, and R.C. Rostomily*†^2,5#,6#^

Supplementary Materials

Supplementary Figure 1. Layout of device microchannel layer.

Supplementary Figure 2. Characterization of cell growth, death, and proliferation in glioma xenograft slice cultures.

Supplementary Figure 3. Xenograft cellular microenvironment.

Supplementary Figure 4. High-throughput drug screening of U87 glioma and GBM8 glioma stem cell lines.

Supplementary Figure 5. Dose-dependent increase in cell death after cisplatin treatment of U87 slices in culture.

Supplementary Figure 6. Characterization of intracranial xenograft slice cultures derived from U87 or GBM8 cells.

Supplementary Figure 7. Restricted lateral spread of cisplatin-mediated cell death in slice cultures on the device.

Supplementary Table 1. Secondary screen results with U87.

Supplementary Table 2. Secondary screen results with GBM8.

**
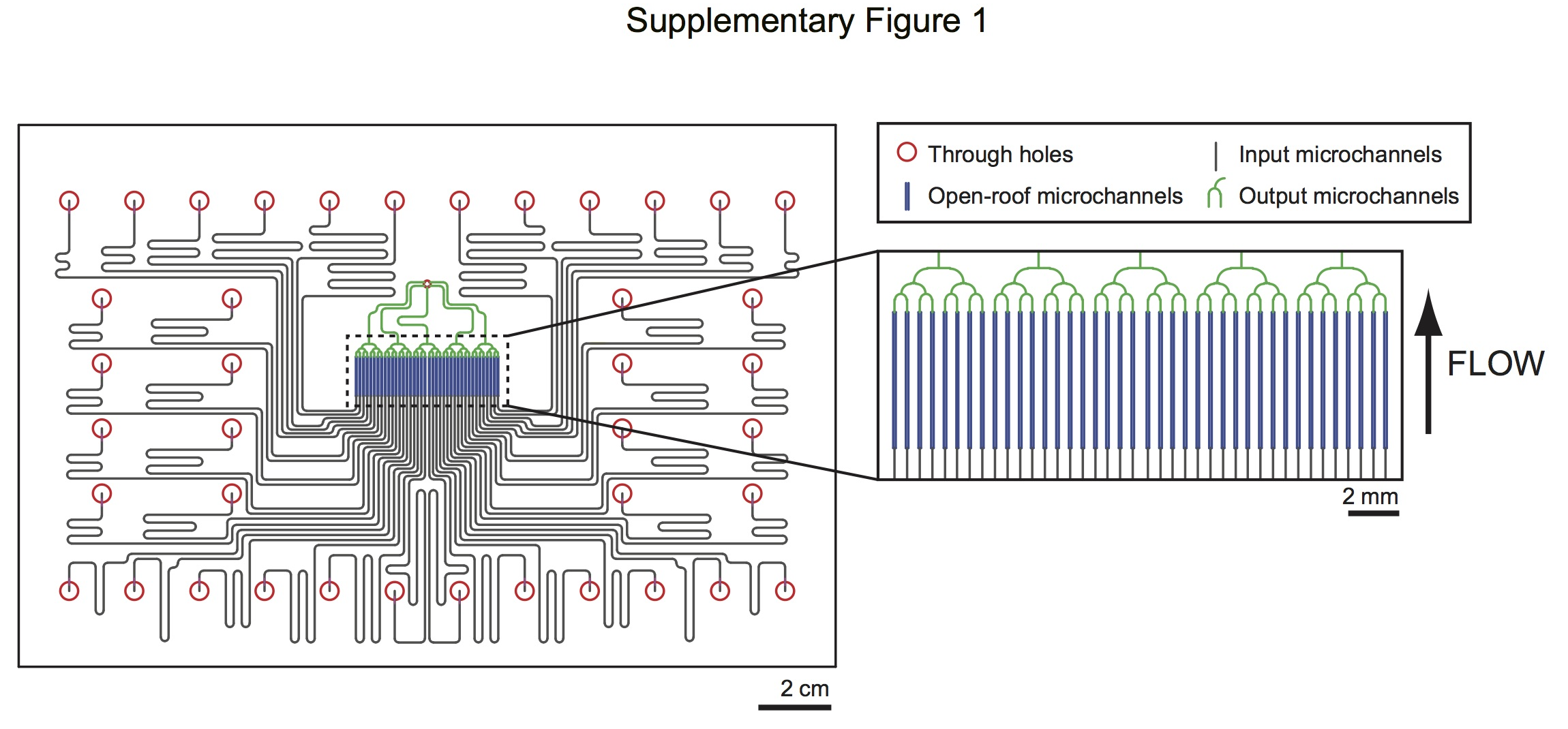
**

**Supplementary Figure 1. Layout of device microchannel layer.** CAD drawings show the device microchannel layer, and the drug delivery area at higher magnification. Through holes (red circles) connect via input microchannels (grey) to the open-roof microchannels (blue), which will be covered and closed by the culture membrane and overlying tissue during device operation. Output microchannels (green) then combine into a single output.


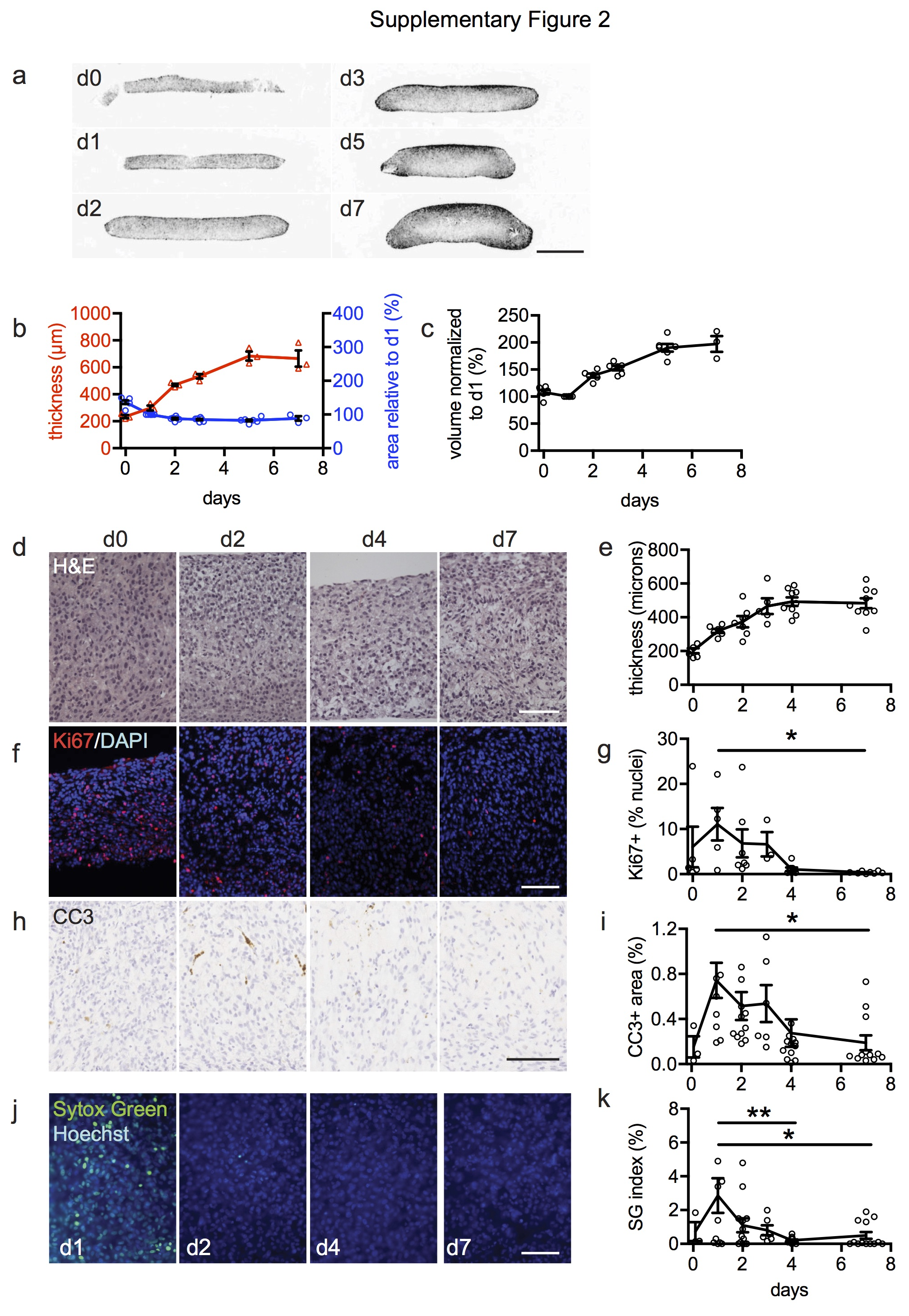


**Supplementary Figure 2. Characterization of cell growth, death, and proliferation in glioma xenograft slice cultures.** U87 flank xenograft slices were cultured for up to 7 days, following by sectioning and staining to quantify slice appearance and cell growth/death. **a-c**) Low-power cross-sections of U87-GFP xenografts (membrane surface down) (**a**) were used to quantify changes in thickness and relative area (**b**) and in volume (estimated by thickness × area) (**c**) over time (n=3 each timepoint). **d-k**) U87 xenograft slices were used to assess overall histological appearance by H&E (D); to quantify changes in slice thickness (**e**, n=6-9 each timepoint); to measure cell proliferation by Ki-67-stained nuclear fraction (**f,g**; n=3-8); and to measure cell death by cleaved caspase 3 (apoptotic cell death; **h,i**; n=3-13) or SYTOX Green versus Hoechst staining (**j**,**k**; n=3-13). Both cell proliferation and cell death peaked on day 1, then declined over the subsequent 3 days in conjunction with a plateau in slice thickness. All curves represent the average ± SEM, one-way ANOVA, Tukey’s multiple comparison test, with * p<0.05, **p<0.01. Scale bar = 500 μm (**a**), 100 μm (**d,f,h,j**).


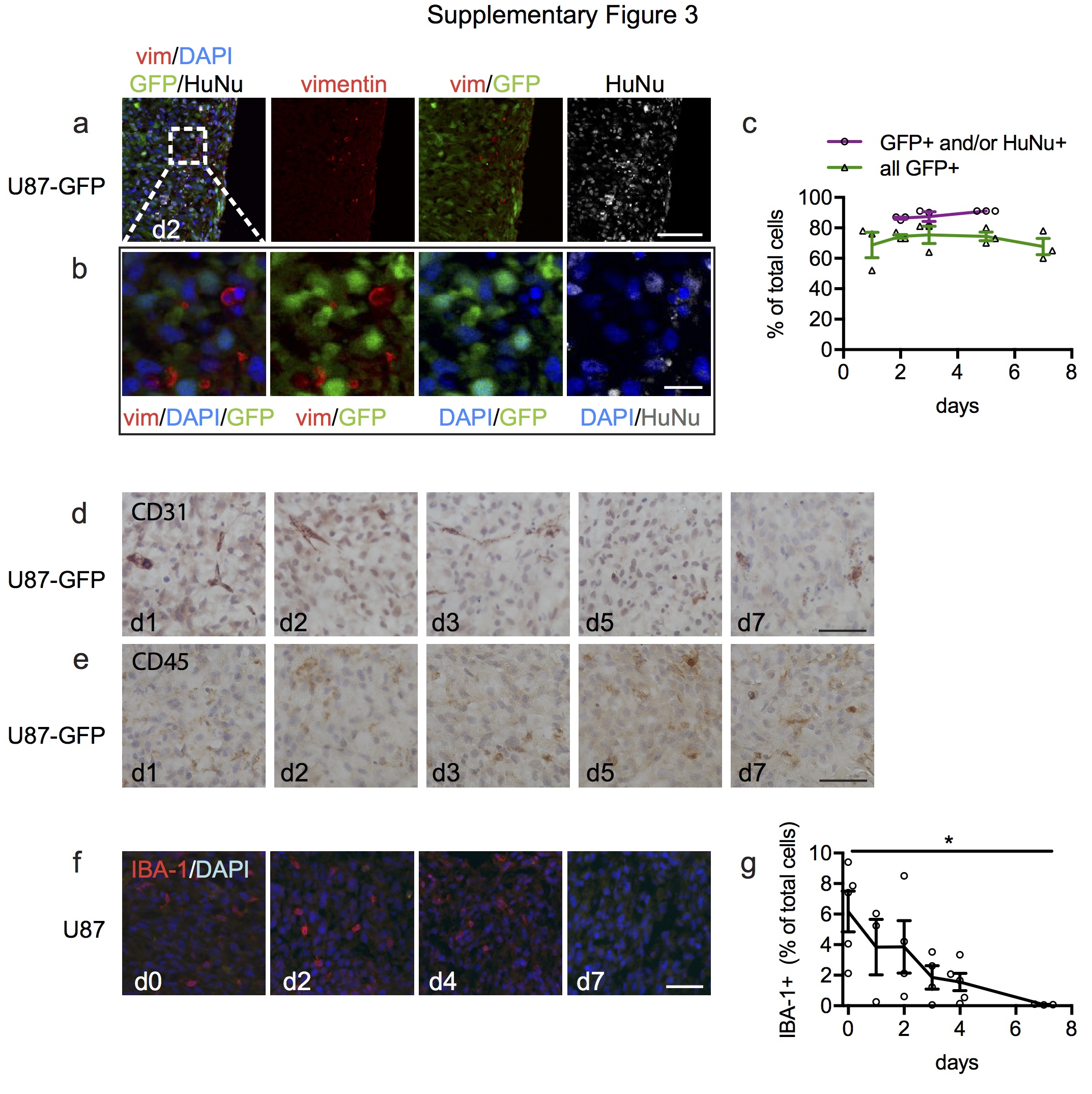


**Supplementary Figure 3. Xenograft cellular microenvironment.**

**a,b**) U87-GFP flank tumor slices were stained after different times in culture with antibodies to human nuclear antigen (HuNu), then confocal-imaged to identify tumor cells (GFP+ and/or HuNu+). The percentage of tumor cells was quantitated as a percentage of total cells (DAPI+) in (**c**) (ave ± SEM, n=3). Fibroblasts were identified in the same sections by antibodies to vimentin to identify mesenchymal stromal cells (vimentin+/GFP-/HuNu-). DAB and hematoxylin immunohistochemistry of the same U87-GFP slices revealed a gradual loss of CD31+ endothelial cells (**d**) and persistence of CD45+ immune cells (**e**). **f,g**) Antibody staining of U87 flank xenograft slices show a decline in IBA-1+ macrophage over 7 days in culture. Average ± SEM, n = 5,3,4,4,5,3. One-way ANOVA, Tukey’s multiple comparison test. * p<0.05 versus d0. Scale bar = 100 μm (**a**), 20 μm (**b**), 50 μm (**d-f**).


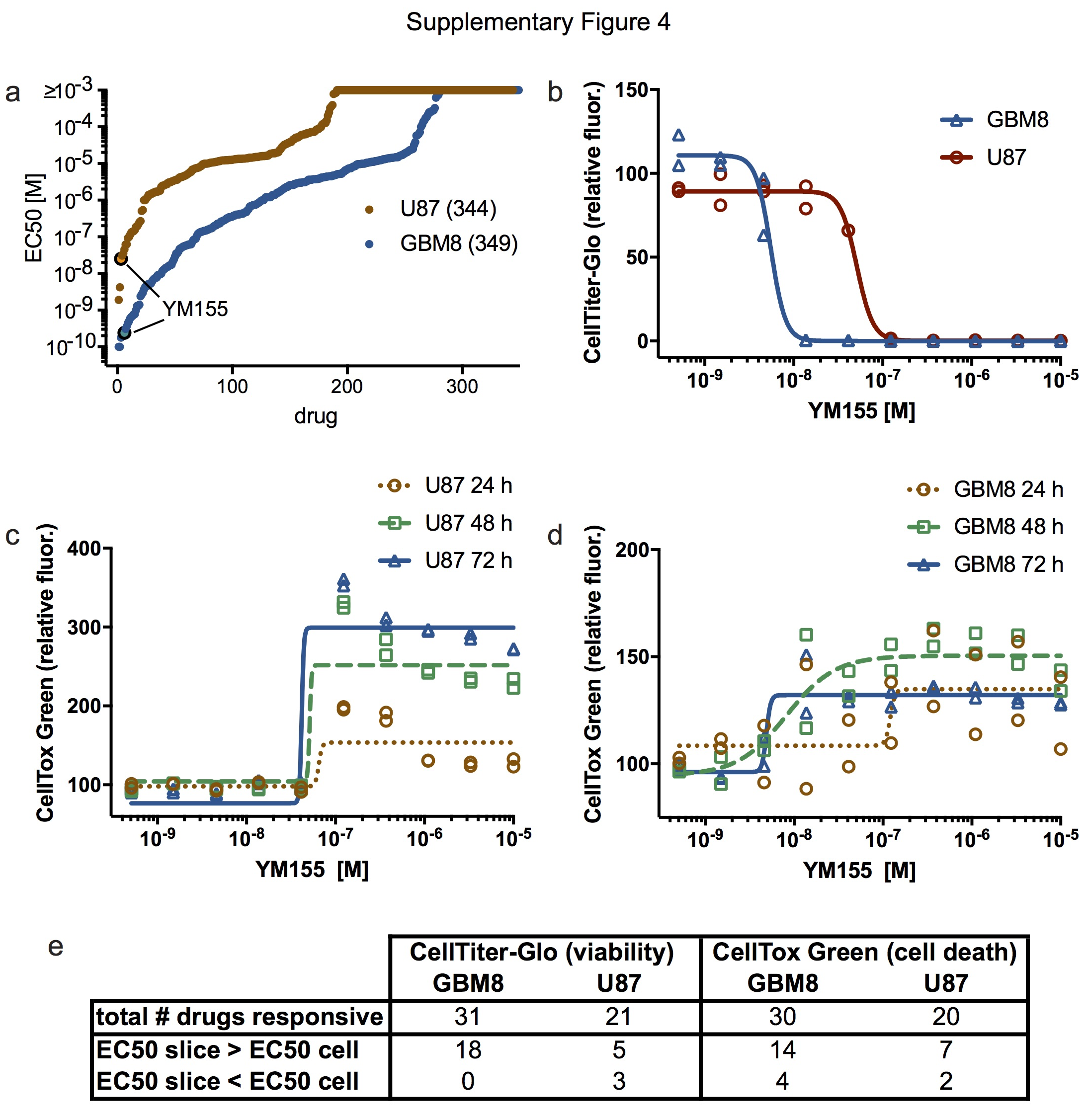


**Supplementary Figure 4. High-throughput drug screening of U87 glioma and GBM8 glioma stem cell lines. A**) Waterfall plots of EC50 values from a primary screen of U87 and GBM8 cells with Selleck anti-cancer library drugs, with reduced viability at 72 h assessed by CellTiter-Glo. The total number of drugs screened (in parentheses) and the position of YM155 are indicated. **B-D**) Example from a secondary screen of U87 and GBM8 cells with 32 drugs, with YM155 dose-dependent growth suppression seen by CellTiter-Glo at 72 h (**B**), as well as a time course of YM155 dose-dependent killing of U87 and GBM8 cells seen by increased CellTox Green fluorescence at 24, 48, and 72 h (**C,D**). **E**) Summary statistics for the ability of the 32 drugs to suppress the growth or viability of U87 and GBM8 cells as a function of culture medium. Cell viability was quantified by CellTiter-Glo and cell death by CellTox Green for each cell type, grown in their routine culture medium (‘cell’) versus in slice culture medium. A ≥4.5 fold increase or decrease in EC50 was used as a cutoff and the number of drugs meeting each condition is specified. Each screen was performed in duplicate.


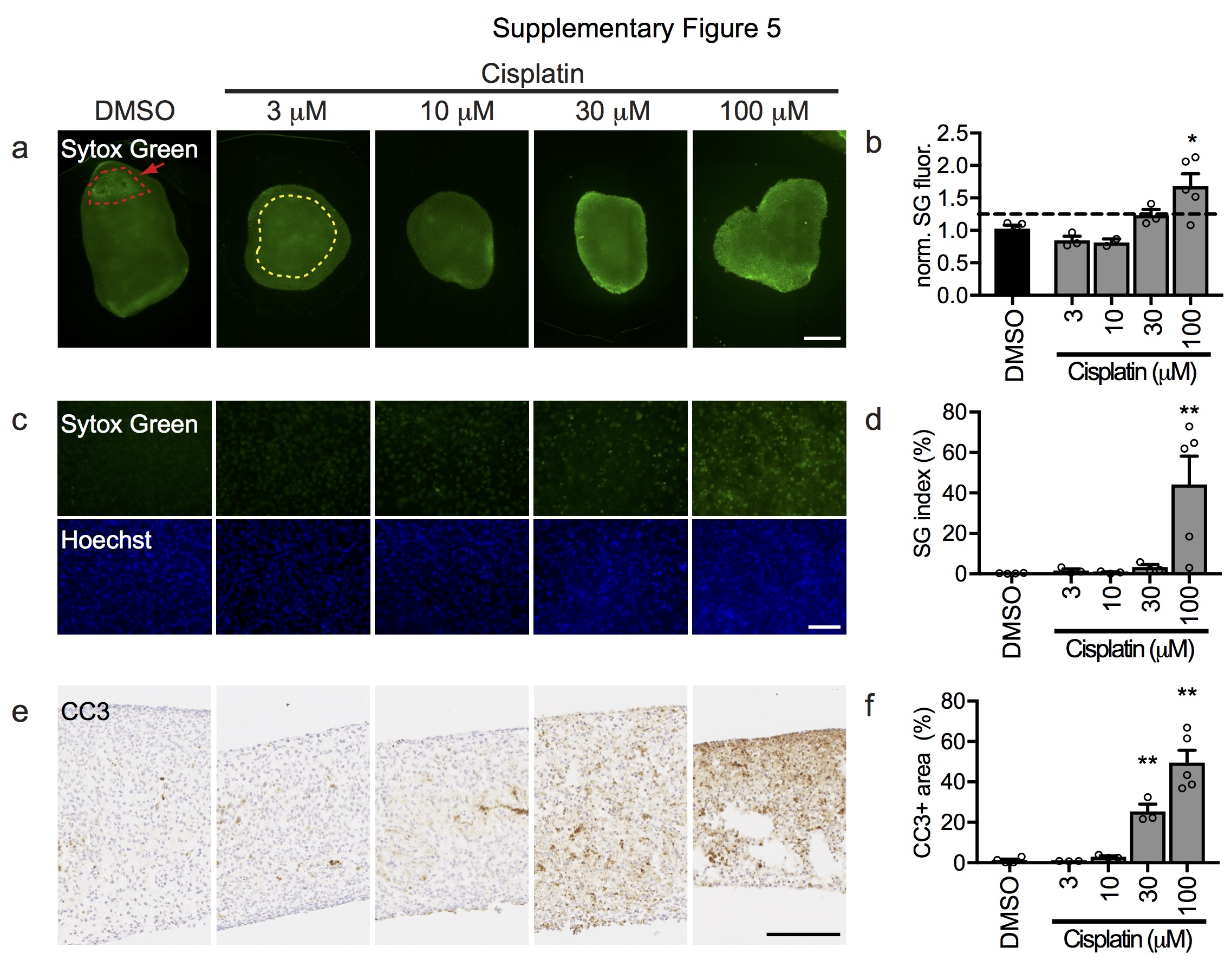


**Supplementary Figure 5. Dose-dependent increase in cell death after cisplatin treatment of U87 slices in culture.** U87 flank tumor slices treated with different doses of cisplatin (CP) from days 1-3 were then exposed to Sytox Green (SG, green dead nuclear stain) and Hoechst (blue nuclear stain) before washing and fixation. Non-specific cell death was assessed in low power images (**a**) as overall fluorescence over the central area (dotted yellow line). A positive control crush lesion on the DMSO slice is outlined in red. Treatment with 100 μM CP led to increased cell death, measured as SG fluorescence normalized to DMSO control (**b**). (**c**,**d**) Analysis of high power images showed a similar increase in cell death at 100 μM CP, calculated as an SG index (% SG+ nuclei/all Hoechst+ nuclei). **e,f**) Apoptotic cell death was assessed by CC3+ immunostaining of cross-sections (**e**) and quantified as CC3+ area (**f**), revealing apoptotic cell death at 30 μM as well as at 100 μM CP. Average ± SEM; n=4,3,3,3,5 (**b,d,f)** except n=2 for 10 μM in (**b**). One-way ANOVA versus DMSO with Dunnett’s multiple comparison test. *p<0.05, **p<0.01. Scale bar = 1 mm (**a**), 100 μm (**c**,**e**).


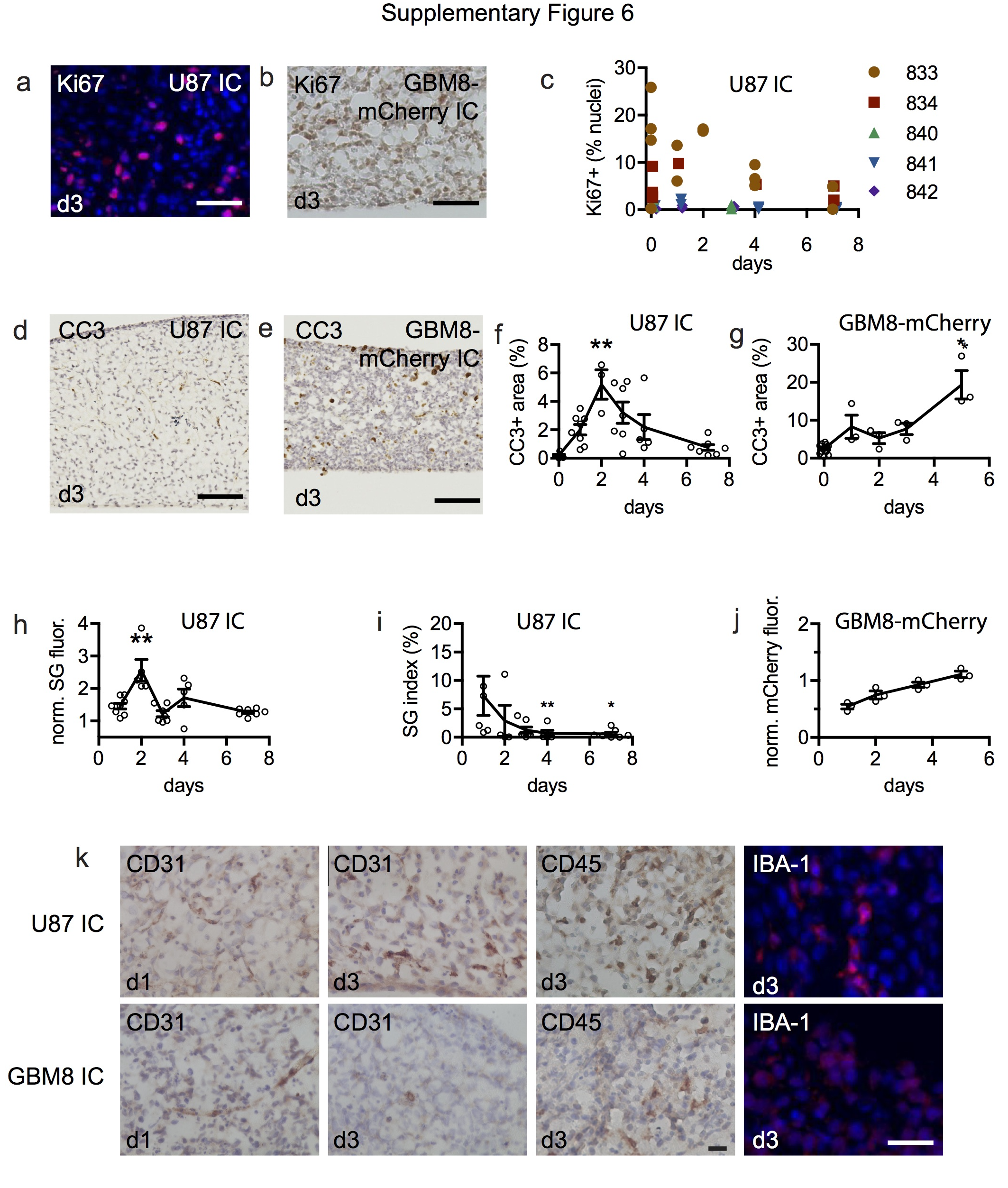


**Supplementary Figure 6. Characterization of intracranial xenograft slice cultures derived from U87 or GBM8 cells. a-c)** Cell proliferation was detected in intracranial xenograft slices by Ki-67/Hoechst (**a**) or Ki-67/ hematoxylin (**b**) staining at d3. **c)** Time course Ki-67 staining as % of total nuclei, shown for individual slices from 5 separate U87-derived xenograft animals (number ID on right). **d-g)** Apoptotic cell death detected by cleaved caspase 3 (CC3) immunostaining in U87 over 7d, or in GBM8-mCherry xenografts over 5d, quantified by measuring CC3+ stained slice area (f,g). Overall cell death was quantified for U87-initiated xenografts by mean SYTOX Green (SG) fluorescence normalized to d3 (**h**), or by an SG index (% SG+ nuclei) (**i**). **j**) mCherry fluorescence (normalized to DMSO controls at d3 run in parallel) in GBM8-mCherry did not decrease over 5d. N = 3 per timepoint. **k**) Presence of additional cell types in U87 and GBM8 slice cultures shown by immunostaining for endothelial cells (CD31), immune cells (CD45), and microglia (IBA-1) at d1 or d3. Graphs show average ± SEM. N=6,8,3,7,5,7 (**f**); n=8,5,7,5,7 (**h**); n=6,4,7,5,7 (**i**); and n=3 for GBM8-mCherry (**g,**j) except for n=14 at d0. One-way ANOVA with Tukey’s multiple comparison test. *p<0.05, **p<0.01 compared to initial timepoint. Scale bar = 50 μm (**a**,**b**), 100 μm (**d**,**e**), 20 μm (**k**).


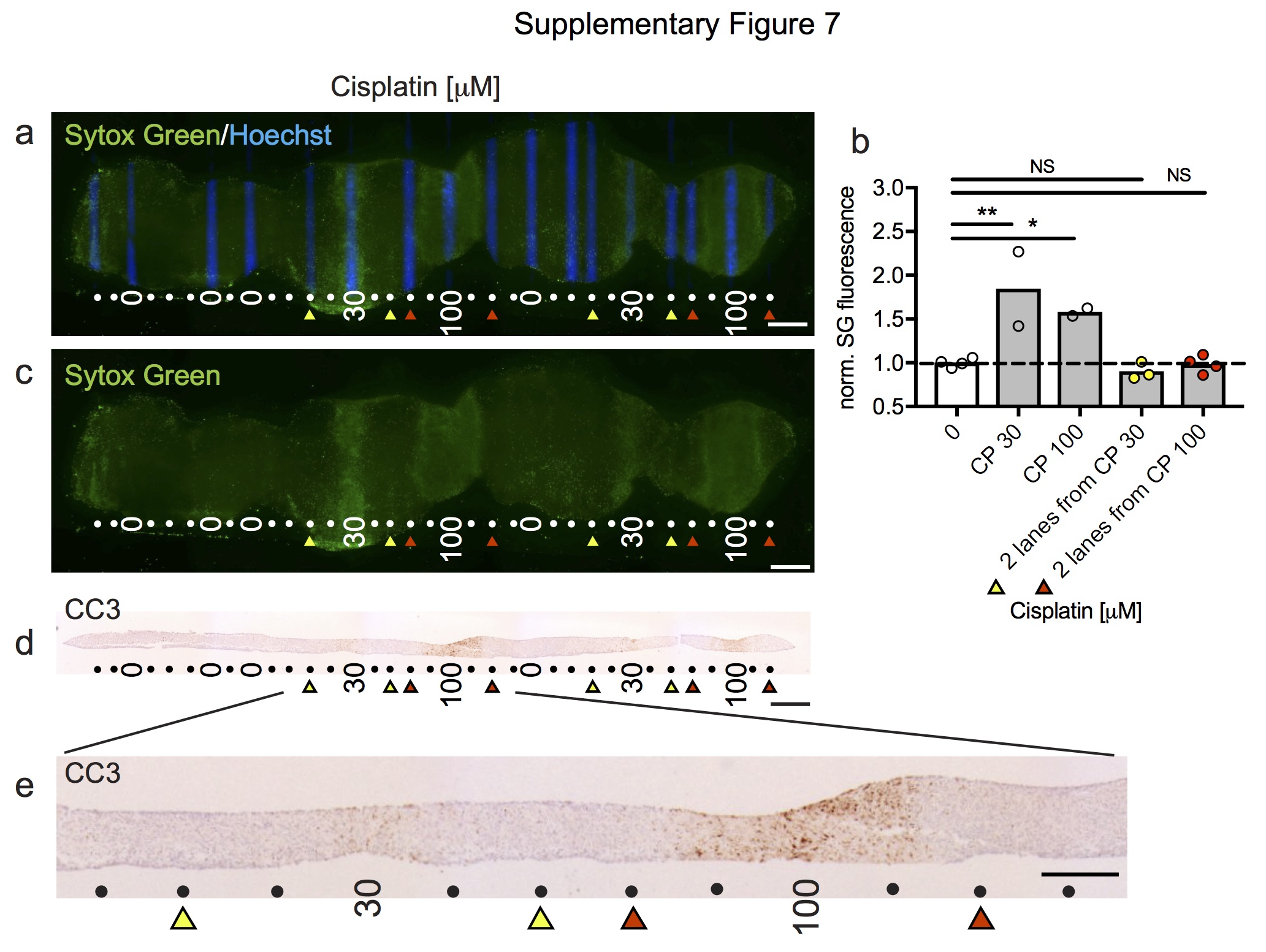


**Supplementary Figure 7. Restricted lateral spread of cisplatin-mediated cell death in slice cultures on the device.** a,c) U87 flank xenograft slices were treated with different cisplatin doses (indicated in μM) for 2 d on-device after 1 d off-device, followed by staining with Hoechst dye delivered through drug lanes and SYTOX Green (SG) staining over the entire slice. Yellow and red triangles identify lanes located 2 lanes over from drug delivery lanes. b) Quantification of cell death by SG fluorescence, normalized to 0 (buffer alone), reveals a significant increase in cell death over drug delivery lanes, but not in lanes located 2 lanes away (identified by triangles as in panels a and c). d,e) Apoptotic cell death, detected by cleaved caspase 3 (CC3) immunostaining, was limited largely to the drug delivery lane with only modest lateral spread at the highest concentration tested (100 μM) that doesn’t reach 2 lanes away. The graph in (b) shows average and individual points. One-way ANOVA versus buffer control, Dunnett’s multiple comparison test. *p≤0.05. **p≤0.01. Individual points and average. N=7,3,4,5,5. Scale bar = 1 mm (a,c,d), 500 μm (e).

**Supplementary Table 1. Secondary screen results with U87.** 3 screen runs (I, II, and III) were performed with U87 cells. D-F12 = U87 cell culture medium. Slice = slice medium. NR = no significant response. ND = no data. Drugs used for slice experiments in bold. CUTOFF: at least ~50 inhibition for CellTiter-Glo, minimum increase of CellTox Green signal for U87 of 200% (~500% max). For slice vs. cell medium (screen III only): red = increase in EC50 of more than 4 fold, blue = decrease in EC50 of more than 4 fold.

**Supplementary Table 2. Secondary screen results with GBM8.** 3 screen runs (I, II, and III) were performed with GBM8 cells. GSC = GBM8 glioma stem cell culture medium. Slice = slice medium. NR = no significant response. ND = no data. Drugs used for slice experiments in bold. CUTOFF: at least ~50 inhibition for CellTiter-Glo, minimum increase of CellTox Green signal for GBM8 of 150% (100-200% max). For slice vs. cell medium (screen III only): red = increase in EC50 of more than 4 fold, blue = decrease in EC50 of more than 4 fold.
